# Overweight and obesity at school entry among migrant and German children: a cross-sectional study

**DOI:** 10.1186/1471-2458-5-45

**Published:** 2005-05-09

**Authors:** Beata Will, Hajo Zeeb, Bernhard T Baune

**Affiliations:** 1 Dep. of Epidemiology & International Public Health, University of Bielefeld, D-33501 Bielefeld, Germany; 2 Dep. of Public Health Medicine, University of Bielefeld, D-33501 Bielefeld. Germany; 3 Department of Psychiatry and Psychotherapy, University of Muenster, D-48149 Muenster, Germany

## Abstract

**Background:**

Overweight and obesity have become a global epidemic and are increasing rapidly in both childhood and adolescence. Obesity is linked both to socioeconomic status and to ethnicity among adults. It is unclear whether similar associations exist in childhood. The aim of the present study was to assess differences in overweight and obesity in migrant and German children at school entry.

**Methods:**

The body mass index (BMI) was calculated for 525 children attending the 2002 compulsory pre-school medical examinations in 12 schools in Bielefeld, Germany. We applied international BMI cut off points for overweight and obesity by sex and age. The migration status of children was based on sociodemographic data obtained from parents who were interviewed separately.

**Results:**

The overall prevalence of overweight in children aged 6–7 was 11.9% (overweight incl. obesity), the obesity prevalence was 2.5%. The prevalence of overweight and obesity was higher for migrant children (14.7% and 3.1%) than for German children (9.1% and 1.9%). When stratified by parental social status, migrant children had a significantly higher prevalence of overweight than German children in the highest social class. (27.6% vs. 10.0%, p = 0.032) Regression models including country/region and socioeconomic status as independent variables indicated similar results. The patterns of overweight among migrant children differed only slightly depending on duration of stay of their family in Germany.

**Conclusion:**

Our data indicate that children from ethnic minorities in Germany are more frequently overweight or obese than German children. Social class as well as family duration of stay after immigration influence the pattern of overweight and obesity in children at school entry.

## Background

Overweight and obesity are rapidly increasing among children and adults. In 1998, the World Health Organisation recognized obesity as a major worldwide public health epidemic [1]. The most recent World Health conference at Geneva called for specific action to halt this epidemic [2].

Overweight and obesity can lead to a wide array of health and social consequences. Childhood overweight appears to be associated with cardiovascular risk factors such a high blood pressure, hyperlipidemia, elevated insulin levels [3] and non-insulin-dependent diabetes mellitus (type 2 diabetes). Other comorbidities include asthma and orthopedic problems as well as a varity of more rare disorders [3,4]. Ultimately overweight in childhood is associated with premature mortality especially if combined with intrauterine growth retardation (IUGR). The psychological well-being and the quality of life can also be affected [3,5,6].

Furthermore overweight and obesity in childhood, particulary in adolescence tends to persist into adulthood. The risk of adult overweight is about twofold increased for individuals who were overweight as children compared with individuals who were not overweight [7,8]. Adulthood overweight are at increased risk of dyslipidaemia, hypertension and type 2 diabetes even if the extra weight was lost during adulthood [6]. Psychological consequences include social and psychological stress, with increased risk of negative self- esteem, social isolation and negative influence on the career and family incomes [7].

There are wide geographical variations of overweight. Comparing reported prevalences of childhood overweight in Europe, Lobstein et al. [9] point out that children residing in central and Eastern Europe have a lower level of overweight than children from other parts of Europe, especially from Southern Europe. Average prevalences in Eastern Europe range from 10% to 18% among children aged 7 to 11 years, whereas values around 20% to 35 % have been reported from countries like Greece and Spain [9,10]. It is suggested that the low obesity prevalence in Eastern Europe is a consequence of the huge economic burden and the associated poverty following the political transition in the 1990s.

Studies in industrialized countries show that children from families with lower socio-economic status particularly suffer from excess weight [11,12]. In less industrialized societies excess weight is found predominantly among children from families with higher socio-economic status [9].

The process of migration, with its economic, social and environmental consequences may also affect health and body weight among migrants. Several US studies show differences in body mass index (BMI) among ethnic minorities [13,14] but empirical data on BMI in children among different ethnic groups in Europe are scarce. In Germany children from non-German ethnic groups nowadays make up a large percentage of children overall. In some cities up to 50% of all children entering school may be first, second or third generation migrants [15]. There are several studies or health reports on overweight and obesity among German and migrant children at school entry [16-19]. Migrant children were consistently found to be more frequently overweight as compared to German children. In Berlin, overweight and obesity was noted as a particular problem among children of Turkish origin [20]. However, since the assessment of migration status is not uniform, comparisons between studies are difficult. In addition, family socioeconomic status and duration of migration are not routinely evaluated. We therefore investigated the prevalence of overweight and obesity among migrant and German children at school entry and to assess the influence of duration of migration and socioeconomic status on overweight and obesity in childhood. It should be noted that the primary aim of the study was to assess differences in health status and health service usage among Germants and Migrants and the current analysis therefore has exploratory character.

## Methods

In 2002, a cross-sectional study was conducted at 12 primary schools in Bielefeld, a town of some 320.000 inhabitants in North-West Germany. The study population comprised of all children registered at these schools for school entry in 2002. Through the city public health office these children and their parents were invited to attend the compulsory medical examination taking place at the local school. The 12 schools (out of a total of some fifty primary schools in the town) were pragmatically selected based on information available for the preceding year. The aim was to include schools with a relatively balanced mix of migrant and German children and fair variation of social status among the population in the respective school districts.

Social and demographic information relevant to the children were derived from a 36 item-questionnaire filled out by parent or relative who accompanied the children [21]. When two adults accompanied the child we collected interview data from only one parent or relative, usually the mother. The questionnaire was available in 4 languages (German, Turkish, Russian, Polish). Parents or relatives were interviewed on the day of the school health examination with the assistance of trained interviewers.

The medical examination of the children was carried out by medical staff from the public health office in Bielefeld using a standardized model of medical school health examination [22]. During the physical and psychological examination, height and weight of children were recorded. All anthropometric measurements of the children were performed in the morning. Body weight was determined to the nearest 500 g using electronic or mechanic non -calibrated scales. During the measurements the children were dressed in light indoor clothes without shoes. No adjustments were made for clothing. Height was measured to the nearest 10 mm using a mobile scale.

657 children aged 6 and 7 appeared in the schools for pre-school examination. Interview data were collected from 565 adult companions (response rate of 86%). The sample size was further reduced to 537 data sets due to merging errors and missing medical data for some children. We also excluded all 12 children who had non-parental companions at the examination.

The statistical analyses were performed on merged data from 525 children (274 boys and 251 girls) and their parents, corresponding to an overall response rate of 79.9% (525/657). 267 (50.9%) parents were of German descent and 258 (49.1%) of Non-German descent. There were 240 females and 27 males among the German parents and 191 females and 67 males among the Non-German parents. Migrant status of the children was based on the migrant status of the interviewed parent. Parents who themselves or whose parents were born in Germany were classified as German, all others were defined as migrants.

The study population consisted of three main migrant groups: 36% of migrants were of Turkish origin, 25% originated from Russia and 15% from Poland. A total of 26 different countries of origin was noted.

A social class index consisting of three social classes (low, medium, high) was constructed using four indicators (primary qualification, professional education, employment status and size of household). We divided these variables into 3 levels (variable professional education in 2 levels) and scored 1–3 points. The sum of the points was than divided in 3 social classes (high social class 10–11 points, medium social class 7–9 points, low social class 4–6 points) [23].

BMI was calculated as body weight divided by body height squared (kg/m^2^). Overweight and obesity were defined according to the recommendations of the International Obesity Task Force IOTF, using international reference values based on data from six countries [24]. These age and sex specific BMI cut-off points for overweight and obesity in children (between 2 and 18 years) were constructed using dataset specific centiles corresponding to the widely accepted adult cut-points of a BMI of 25 kg/m^2 ^(overweight) and 30 kg/m^2 ^(obesity).

For the calculation of overweight and obesity the exact age of the children was rounded to the nearest half year. Children with BMI at or above cut-off value corresponding to a BMI of 25 kg/m^2 ^in late adolescence (e.g. BMI of 17.55/17.34 for 6 year old boys/girls) were classified as overweight (or obese when the respective cut-points were reached). For comparative purposes, we also used national BMI reference curves for Germany [25] in some instances. Here, the age-and sex-specific 90^th ^and 97^th ^BMI percentile are used as cut-point for overweight and obesity, respectively.

### Statistical analysis

The analysis was performed on the data set containing merged data from both the parental survey and childrens' medical examination.

We used descriptive statistical methods to compare obesity and overweight between migrant and German children. Data were stratified according to migrant status, social status and duration of stay in Germany. Differences in proportions were tested by Fisher's exact or Chi-square test, trends in the prevalence of overweight were analysed with chi-square trend test.

Logistic regression models were used to simultaneously analyse the association between overweight and gender, social status, ethnic background and duration of stay in Germany among migrants. Adjusted odds ratios and 95% confidence intervals were calculated. The statistical analysis was performed with the software package SPSS (version 11.5; SPSS Inc, Chicago, IL).

## Results

In 2002 the overall point prevalence of overweight (including obesity) in examined children starting primary school was 11.9%. 2.5% of children were obese according to the criteria mentioned above. More girls than boys were overweight (15.3% vs. 8.8%; p = 0.03) and obese (4.0% vs. 1.1%; p = 0.046). Comparing German and children from ethnic minorities the prevalence of overweight as well as of obesity was higher in migrant children in both genders. Table 1 summarizes the results.

**Table 1:** Prevalence of overweight and obesity^# ^of children by gender and migrant status

		Migrants N = 258		Germans N = 265		p – value*	
		
		overweight	obesity	overweight	obesity	overweight	obesity
All	n/n_Total_ %(95% CI)	38/258 14.7 (10.4–19.1)	8/258 3.1 (1.0–5.2)	24/265 9.1 (5.6–12.5)	5/265 1.9 (0.2–3.5)	0.058	0.413
Boys	n/n_Total_ %(95% CI)	13/129 10.1 (4.9–15.3)	2/129 1.6 (-0.6-3.7)	11/145 7.6 (3.3–11.9)	1/145 0.7 (-0.7-2.0)	0.524	0.603
Girls	n/n_Total_ %(95% CI)	25/129 19.4 (12.6–26.2)	6/129 4.7 (1.0–8.3)	13/120 10.8 (5.3–16.4)	4/120 3.3 (0.1–6.5)	0.077	0.751

*Fisher's exact test comparing migrants and Germans

^#^Variable BMI contains 2 missing values

When applying national German BMI references [25], slightly lower prevalence estimates were calculated. Still, the point prevalence of overweight in migrant children was significantly higher compared to German children (12.0% vs. 5.7%; p = 0.01) while differences in obesity prevalence were not significant (3.5% vs. 2.3%). When stratified by gender, only migrant girls were significantly more overweight than their German peers (p = 0.04). Children from families with low socioeconomic status (SES) were significantly more often overweight (18.9%) than children with average (8.9%) or high SES (14.7%) (p = 0.02). Among Germans the prevalence was highest among children from low SES families (23.5%). Conversely the prevalence of overweight was highest among migrant children with high SES (27.6%) (Table 2).

**Table 2:** Overweight by migrant status and social class

		Migrants N = 240	Germans N = 256	p – value*
	
Social class^#^		Prevalence of overweight		
Low	n/n_Total_ %(95% CI)	14/78 17.9 (9.4–26.5)	4/17 23.5 (3.4–43.7)	0.733
Medium	n/n_Total_ %(95% CI)	14/133 10.5 (5.3–15.7)	12/159 7.5 (3.4–11.7)	0.414
High	n/n_Total_ %(95% CI)	8/29 27.6 (11.3–43.9)	8/80 10.0 (3.4–16.6)	0.032

*Fisher's exact test comparing migrants and Germans

^#^social class index using primary educational qualification, professional education, employment status and size of household (27 missing values). Variable BMI contains 2 missing values

On average, children from migrant families had a lower socio-economic status when compared to German children (p < 0.001). Further analyses of socioeconomic status among migrants from different countries and regions of origin indicated that on average Turkish children were of lower social status than children from Eastern European families (Data not shown).

Nevertheless, there were no differences in overweight prevalence when comparing children according to country/region of origin (Turkey, Eastern Europe/Russia, others) (Table 3).

**Table 3:** Overweight by ethnic origin of family

		Ethnic origin			
		Turkey	Eastern Europe/Russia	Other	All
Prevalence of overweight	n/N %(95% CI)	13/89 14,6 (7,3–21,9)	19/126 15,1 (8,8–21,3)	6/43 14,0 (3,6–24,3)	38/258 14,7 (10,4–19,1)

In the bivariate analysis we found slight differences in the patterns of overweight among migrants depending on duration of stay in Germany. The results indicated that the prevalence of overweight among migrant children was highest in the category of highest family duration of stay in Germany (20.9% versus 12.9% in those staying 1–9 years). However, the trend across categories was not significant (p = 0.32).

In the multivariate analysis (Table 4) long duration of stay of the family in Germany (30+ years) was associated with overweight in migrant children (OR = 1.92; 95% CI = 0.97–3.79). Migrant girls had an increased risk of overweight as compared to boys. Odds ratios for migrant children with low and high SES were similar, whereas a medium SES was associated with a decreased risk of overweight in the study. In a second model including only socioeconomic status and country/region of origin, results for SES remained unchanged. None of the odds ratios for specific country/region of origin adjusted for SES were significantly different from 1, using Germany as reference.

**Table 4:** Results of the logistic regression model of risk factors for overweight in migrant children

	**OR**	**95%CI**	**p-value**
Boys*	1		
Girls	2.19	0.98–4.92	0.06
social class			
Low *	1		
Medium	0.57	0.33–0.97	0.04
High	1.63	0.83–3.22	0.16
origin			
Turkey*	1		
Eastern Europe	1.15	0.65–2.04	0.63
other	0.92	0.45–1.87	0.81
Duration of stay (years)			
1–9 *	1		
10–19	0.89	0.48–1.67	0.74
20–29	0.95	0.47–1.92	0.88
30+	1.92	0.97–3.79	0.06

*Reference category

## Discussion

The present cross-sectional study shows that the prevalence of overweight and obesity was slightly higher among migrant children than among German children. Socioeconomic status seems to be associated with overweight and obesity in migrant children at school entry whereas duration of stay after immigration to Germany shows no clear pattern of association. Other studies in Germany have reported more marked differences between German and migrant children with regard to overweight and obesity. Among boys in Bavaria, Kalies [26] found that the frequency of overweight and obesity in non-German compared to German children was 1.9 times higher for overweight and 2.4 times higher for obesity. Similar findings have been reported from Berlin [20] and the federal state of Lower Saxony [27]. For Northrhine-Westfalia where the city of Bielefeld is located, only basic data are available showing similar patterns but an overall much lower overweight prevalence of 7% or less in all groups [28] while older data from Dortmund, a larger but structurally comparable town, show an overweight prevalence of about 12% among second generation migrant children [19]. Results of our study are consistent with these data, although the smaller sample size affects discriminatory statistical power. The choice of reference and the definition of migrant status (see below) may also contribute to the slightly different results.

In Europe, only a few studies on Body Mass Index (BMI) examined the potential impact of ethnicity or migration status on overweight and obesity. A recent UK survey [29] showed that Indian and Pakistani boys had a higher prevalence of overweight compared with boys in the general UK population while Bangladeshi and Chinese boys had a lower overweight prevalence. Among girls, Afro-Caribbean and Pakistani girls more frequently were overweight while Indian and Chinese girls had a lower overweight prevalence compared to girls from the general population. In France, the children of Maghrebian immigrants were more obese than French children in cross-sectional surveys conducted in the 1970ies and the 1990ies [30]. The overall prevalence of obesity increased from 8% to 13% over this period. Data from health surveys in 1992/93 and 1993/94 among children in the Netherlands showed that the average body mass index was higher among Turkish and Moroccan children than among Dutch children [31].

Geographical differences in overweight in Europe were demonstrated by Lobstein et al. [9]. Children from countries in Central and East Europe generally showed a low prevalence of overweight. On the other hand, a higher prevalence of overweight was found in children in Southern European countries. Recent data from Edirne in Turkey demonstrate comparatively low prevalences of overweight and obesity among adolescents [32].

The international variability of definitions concerning paediatric overweight and obesity implies methodological difficulties when comparing prevalences internationally. In adults, the BMI cut-points of 25 and 30 are widely used to define overweight and obesity. Unlike in adults, BMI in children varies substantially by age and gender during childhood and adolescence. Unfortunately no commonly accepted standard has yet emerged. Different reference systems have been proposed and these references vary considerably. International sex – and age -specific cut-points proposed by the IOTF [24] were used in the present study in order to allow national as well as international comparisons. The IOTF reference has attracted some criticsm, mainly based on the argument that data from only six countries form the basis of the international reference, thus putting representativity on a world scale in doubt [33]. It appears disputable whether the use of national reference data is preferable when assessing overweight and obesity among migrant children. The comparison to reference curves from the country of origin would disregard the environmental (e.g. nutritional) changes associated with migration in many instances. On the other hand, using a German national reference is also not fully satisfactory since differences in lifestyle or genetically determined factors related to body composition might persist for generations. An international standard appeared the most suitable option in this situation. Undoubtedly, the arbitrary choice of cut-off points and other problems associated with BMI-based classification systems in childhood will require further debate [34-36].

Our results do not clearly show an impact of family duration of stay after immigration (prior to 1980) into Germany on overweight. However, a long duration was associated with relatively higher prevalences of overweight and obesity. Adaptation and assimilation as well as (modestly) growing wealth of migrants in Germany after decades of life in Germany could partially explain these findings, in particular since the overweight was highest among well-off children. An association of overweight among migrant children with a long duration of stay in Germany has not been reported previously. A period effect due to social and political circumstances as a potential explanation needs to be considered. The possible impact of such changes, however in opposite direction, can be more clearly seen in data from Russia. The prevalence of overweight in Russia decreased from 26.4% in 1992 to 10.2% in 1998 in 6–9 y-old children [35] and the prevalence of underweight in children rose during the same time [9]. Economical recession during this time is a plausible explanation of this finding. In Poland, Koziel et al. found a slightly decreasing trend in overweight between 1987 and 1997 in 14y-old boys [37].

In general, studies on socioeconomic status and overweight suggest that overweight is more prevalent among children of low income families in developed countries. In contrast, the vast majority of studies in developing countries show a positive relationship between socioeconomic status and obesity among children [9,38]. Our study, carried out in a developed country, generally shows consistent results since overweight was more frequent among lower class children. However, among children from families with a non-German origin, the highest prevalence of overweight was observed in children in the high social class. It should be noted, however, that misclassification of socioeconomic status cannot be ruled out in our study since the classification was based on data supplied by one parent, mostly mothers, only.

Further limitations of our study need to be taken into consideration. We did not collect a representative sample but had to use a more pragmatic approach in selecting the study group. However, the schools included in the study represent a fair cross-section of different primary schools in Bielefeld. The assessment of body weight and height was subject to some variation since identical scales were not available at all school health examinations. All examinations were done by the same three teams of school health personnel. Finally the migrant status of children was based on rather differentiated parental data, different from the usual, but unsatisfactory approach of using nationality as indicator. We favour this more complex approach, but realise that comparisons with other studies may be difficult.

## Conclusion

Our study results underline the importance of overweight and obesity as an important public health issue in young children and highlight patterns of overweight and obesity among native and migrant children in Germany. New studies such as the currently ongoing youth health survey [39] will help to clarify the association of overweight and obesity in children with social class and the duration of residence in the host country after migration. Public health interventions aiming at a change in nutrition and behaviour (sports, healthy life style) are likely to benefit from more detailed knowledge about specific risk groups.

## List of abbreviations

BMI: Body mass index

OR: Odds ratio

CI: Confidence interval

## Competing interests

The author(s) declare that they have no competing interests.

## Authors' contributions

HZ and BB planned and co-ordinated the study. BW participated in field work, managed the data and performed the statistical analysis. All authors jointly wrote the paper.

## Pre-publication history

The pre-publication history for this paper can be accessed here:


